# Endogenous Galectin-9 Suppresses Apoptosis in Human Rheumatoid Arthritis Synovial Fibroblasts

**DOI:** 10.1038/s41598-018-31173-3

**Published:** 2018-08-27

**Authors:** Mark J. Pearson, Magdalena A. Bik, Caroline Ospelt, Amy J. Naylor, Corinna Wehmeyer, Simon W. Jones, Christopher D. Buckley, Steffen Gay, Andrew Filer, Janet M. Lord

**Affiliations:** 1 Aston Medical School, Aston University, Birmingham, B4 7ET UK; 2 NIHR Birmingham Biomedical Research Centre, University of Birmingham, Birmingham, B15 2TT UK; 3 Center of Experimental Rheumatology, Department of Rheumatology, University Hospital of Zurich, CH-8091 Zurich, Switzerland; 4 MRC-Arthritis Research UK Centre for Musculoskeletal Ageing Research, Institute of Inflammation and Ageing, University of Birmingham, Birmingham, B15 2TT UK

## Abstract

Galectin-9 (Gal9) has been postulated to have anti-inflammatory properties based on the ability of exogenous Gal9 to induce apoptosis in synovial fibroblasts in animal models of rheumatoid arthritis (RA). Here we aimed to assess the potential role of endogenous Galectins, including Gal9, in the inflammatory pathology of the RA synovium in humans. Firstly expression of Galectins 1–9 was determined in synovial fibroblasts (RASF) and dermal fibroblasts (DF) isolated from RA patients, the latter representing a non-inflamed site. We then further challenged the cells with pro-inflammatory TLR agonists and cytokines and assessed Galectin expression. Gal9 was found to be differentially and abundantly expressed in RASF compared to DF. Agonists of TLR3 and TLR4, along with IFNgamma were also found to induce Gal9 expression in RASF. siRNA was then used to knock-down Gal9 expression in RASF and the effects of this on apoptosis and cell viability were assessed. Increased apoptosis was observed in RASF following Gal9 knock-down. We conclude that, unlike exogenous Gal9, endogenous Gal9 is protective against apoptosis and enhances synovial fibroblast viability suggesting that its role in RA is both pathogenic and pro-inflammatory.

## Introduction

Rheumatoid arthritis (RA) is a chronic inflammatory disease which leads to destruction of the joint and expansion of the synovial membrane leading to an “activated” synovial membrane. Multiple cell types are recruited to the inflammatory milieu which develops within the normally acellular synovium. Although the exact mechanisms driving the onset and maintenance of inflammation in RA are still being determined, it is known that fibroblasts, macrophages and pro-inflammatory B and T cell populations significantly contribute to RA disease pathology^1–6^ including IL-6, IL-23p19, CCL-20, and GM-CSF^7–9^. Their presence within the RA synovium is thus thought to be an important driver of the chronicity of inflammation^1,10–12^.

Galectins are an evolutionarily conserved family of immunomodulatory animal lectins which are expressed in a number of immune cell populations including macrophages, T cells and fibroblasts^13^. Eleven galectins have been described in humans and they have a broad range of actions^14^. In addition, several galectins have been implicated in the regulation of cell death, notably galectins -1 (T and B lymphocytes), -7 (keratinocytes and carcinomas), -8 (carcinomas), -9 (thymocytes) and -12 (adipocytes) (reviewed in^15^). Galectin-9 (Gal9) was first described as a selective chemoattractant for eosinophils^16^, but is now known to have a wider function including as a urate transporter in the kidney^17^. Gal9 functions as a voltage-sensitive channel that mediates transport of urate, a product of purine metabolism that is elevated in the serum of individuals with renal dysfunction and associated with development of gout.

In the immune system Gal9 has been shown to regulate interactions between thymic epithelial cells and thymocytes and promotes apoptosis of immature thymocytes when applied exogenously^18^. Similar to galectin-1, Gal9 induces cell death of mature activated T cells through caspase and calpain-dependent pathways^19^. The surface receptor that is crucial for exogenous Gal9 function is TIM3 (T cell immunoglobulin- and mucin domain containing molecule) expressed on differentiated T helper cells^20,21^. Dysregulation of this signalling pathway has been documented in autoimmune diseases such as multiple sclerosis^22^. Activation of the Gal9/TIM3 pathway has also been shown to be important in graft rejection^23^. In addition to pro-apoptotic activity, Gal9 induces differentiation of naïve T cells into a regulatory phenotype while inhibiting their development into Th17 cells^2^ and stimulates maturation of dendritic cells with production of Th1-type cytokines^24^. Gal9 has been shown to be expressed in RA synovium and it has been reported that a mutant form of Gal9 was able to induce apoptosis in synovial fibroblasts when added exogenously to cell cultures^25^. Furthermore, Gal9 was shown to have a beneficial effect on mouse collagen-induced arthritis when given exogenously^25^. However, it is clear that galectins often have different functions in the extracellular and intracellular context^14,26^ and the role of endogenous Gal9 in synovial fibroblasts and RA pathogenesis has not been established. With an emerging role for galectins in immune modulation and inflammatory diseases the present study examined galectin expression in the specific context of RA.

## Results

### Galectin 9 is differentially expressed in RA synovial tissue

**Figure 1:**
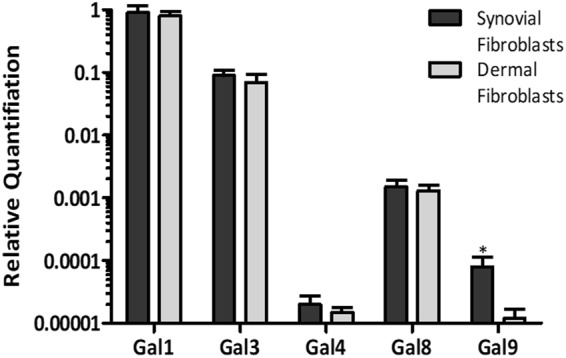
Galectin expression was assessed in synovial and dermal fibroblasts from RA patients by real-time PCR. Data are mean ± SD and * indicates p < 0.05, n = 6.

**Figure 2:**
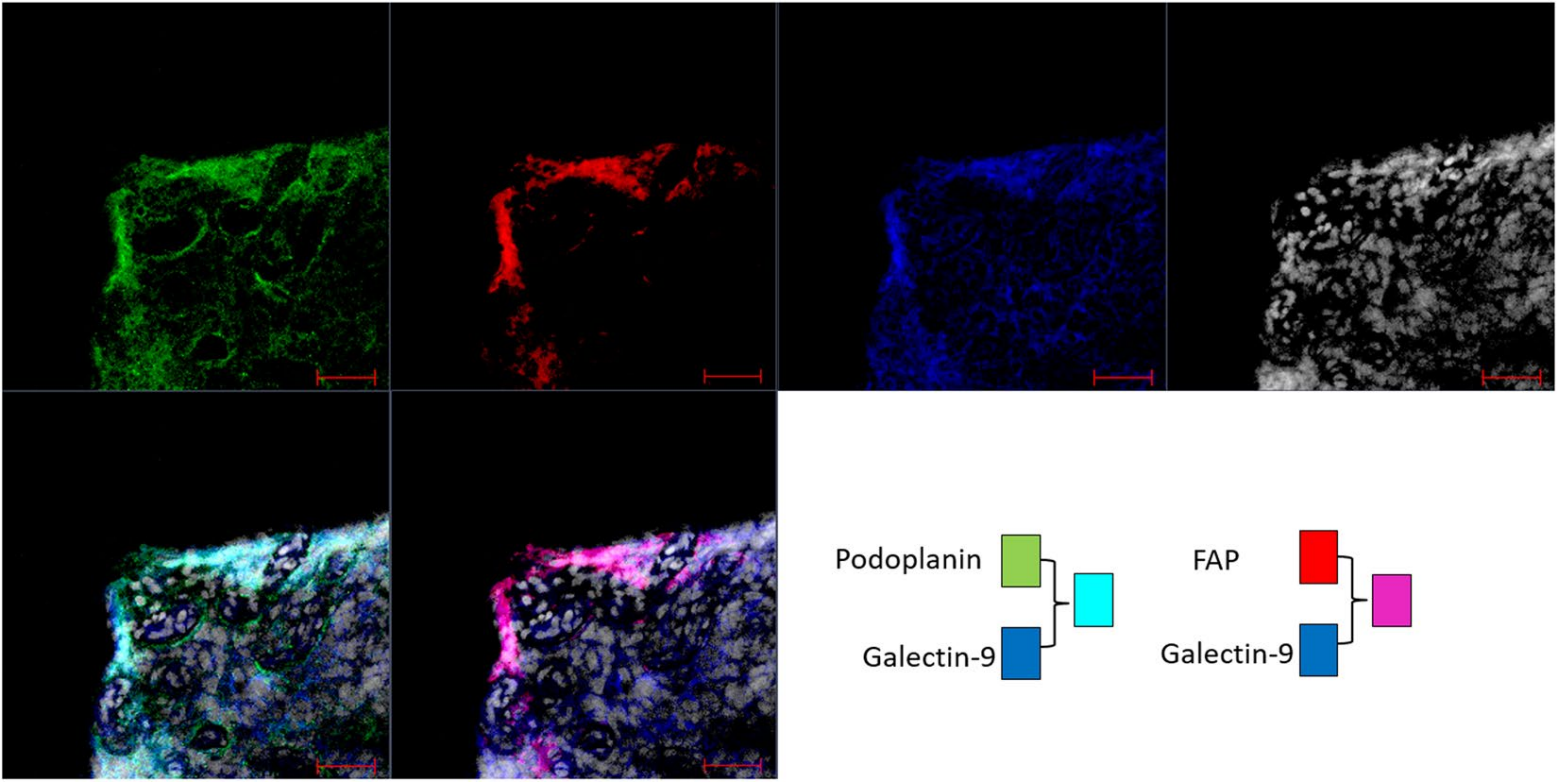
Synovial membrane sections were stained for Podoplanin (green), FAP (red), galectin-9 (blue) and nuclei using DAPI (white). Co-localisation of podoplanin and galectin-9 can be seen as cyan, co-localisation of FAP and galectin-9 appears magenta. Scale bar 50 *μ*m.

Analysis of galectin expression by real time PCR in synovial fibroblasts and matched skin fibroblasts showed expression of Gal1, -3, -4, -8 and -9 in both synovial and dermal fibroblasts (Fig. 1). However, Gal9 was the only galectin that showed increased expression in the synovial compared to the dermal fibroblasts (p < 0.01). Immunofluorescence staining of synovial tissue sections from RA patients confirmed extensive Gal9 expression. Co-staining with the synovial fibroblast markers, podoplanin and fibroblast activation protein (FAP) demonstrated that Gal9 was expressed largely by fibroblasts (Fig. 2). Given these data, we focussed on the regulation and role of Gal9 in synovial fibroblasts.

### Regulation of Gal9 expression

#### Toll-Like Receptor effects on Gal9 Expression

**Figure 3:**
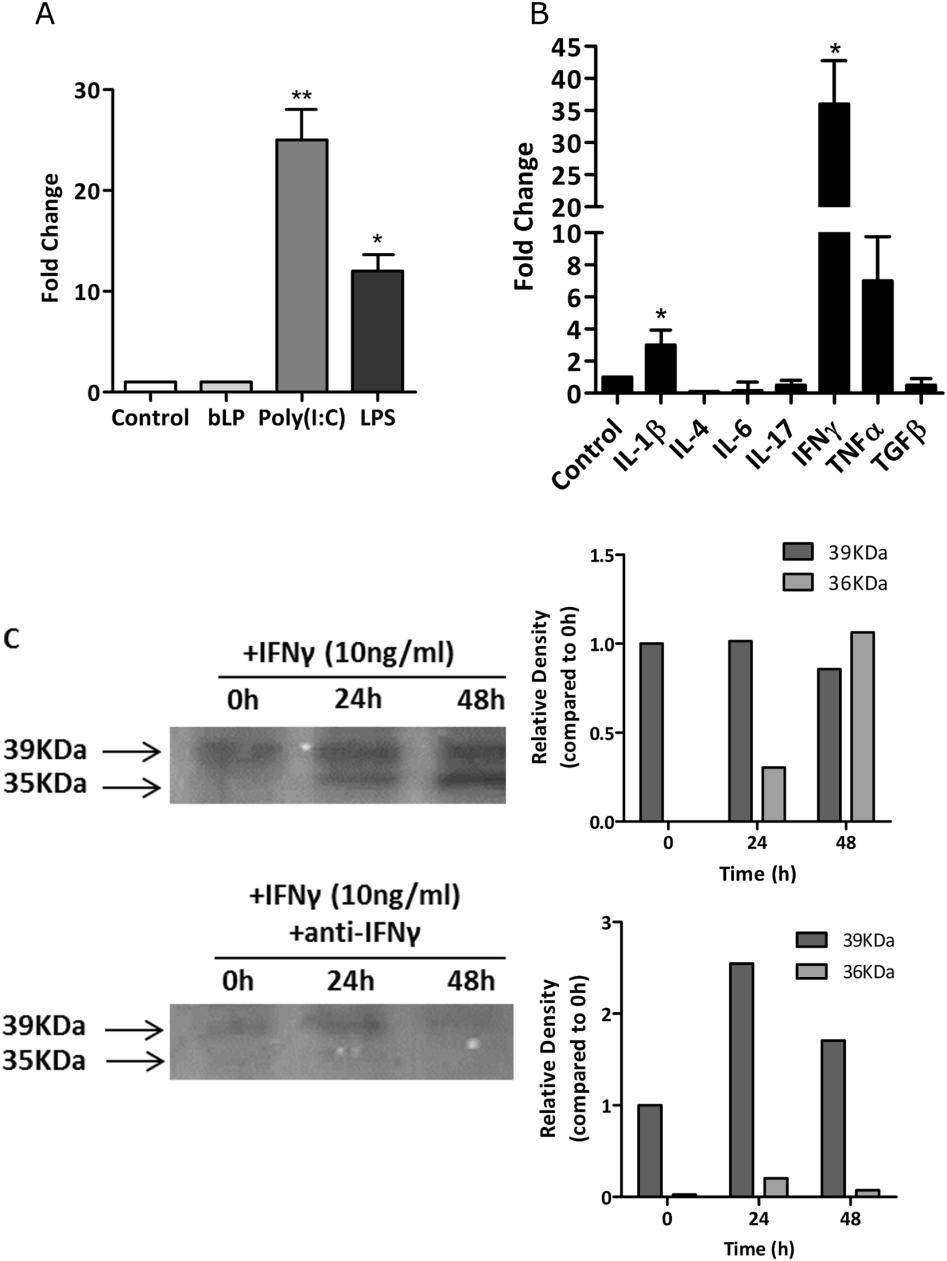
RA synovial fibroblasts were stimulated with (**A**) TLR agonists, TNF*α*, IL-1*β*, bLP, poly(I:C), LPS and (**B**) pro-inflammatory cytokines –IL-1*β*, IL-4, IL-6, IL-17, IFN*γ*, TNF*α* and TGF*β*. mRNA was extracted and fold-changes were plotted. (**C**) The increase in Gal9 expression following IFN*γ* stimulation was confirmed at the protein level by Western blotting. Long (39 KDa) and short (35 KDa) isoforms of Gal9 are shown. The short isoform was induced by IFN*γ* stimulation whereas the long isoform is constitutively expressed. *p < 0.05, **p < 0.01. Full uncropped versions of the blots are shown in the supplementary data.

In order to assess which factors might modulate Gal9 expression we first considered whether toll-like receptor (TLR) activation could affect expression of Gal9. RA synovial fibroblasts were stimulated with the TLR ligands bLP, poly(I:C) and LPS. No significant effects on Gal9 mRNA expression were observed following stimulation with bLP, but stimulation with poly(I:C) (a TLR3 agonist) gave a significant (p < 0.01) 25-fold increase in Gal9 mRNA. LPS (a TLR4 agonist) stimulation led to a smaller, but significant (p < 0.05) 10-fold increase in Gal9 mRNA (Fig. 3A). Thus, Gal9 expression can be induced by activation of the TLR3 and TLR4 pathways. By contrast Gal3 expression could not be induced by any of the TLR ligands used here, suggesting some selectivity for TLR3 and TLR4 signalling towards induction of Gal9 (data not shown).

#### Cytokine effects on Gal9 expression

The RA synovial environment contains many pro-inflammatory cytokines, including IL-1*β*, IL-4, IL-6, IL-17, IFN*γ*, TNF*α* and TGF*β* which mediate different aspects of disease pathology. In order to determine whether they play a role in the upregulation of Gal9 expression, RA synovial fibroblasts and matched skin fibroblasts were stimulated with each of the aforementioned cytokines. Only IL-1*β* (4-fold) and IFN*γ* (35-fold) were found to induce significant (p < 0.05) increases in Gal9 mRNA expression (Fig. 3B). The effects of IFN*γ* were studied further with increases in Gal9 protein expression in synovial fibroblasts in response to IFN*γ* stimulation confirmed by Western blot (Fig. 3C, upper panel). Two isoforms of Gal9 exist -a long (39 KDa) and short (35 KDa) form. The long isoform was constitutively expressed in RA synovial fibroblasts and expression was increased in a time-dependent manor following IFN*γ* stimulation for 24–48 hours. However IFN*γ* stimulation induced a more profound effect on the short isoform, which was not readily detectable in untreated cells but was evident after 24 hours. Increased expression of the short isoform could be inhibited by an anti-IFN*γ* antibody (Fig. 3C, lower panel).

### Effect of Galectin 9 Silencing on synovial fibroblasts

**Figure 4:**
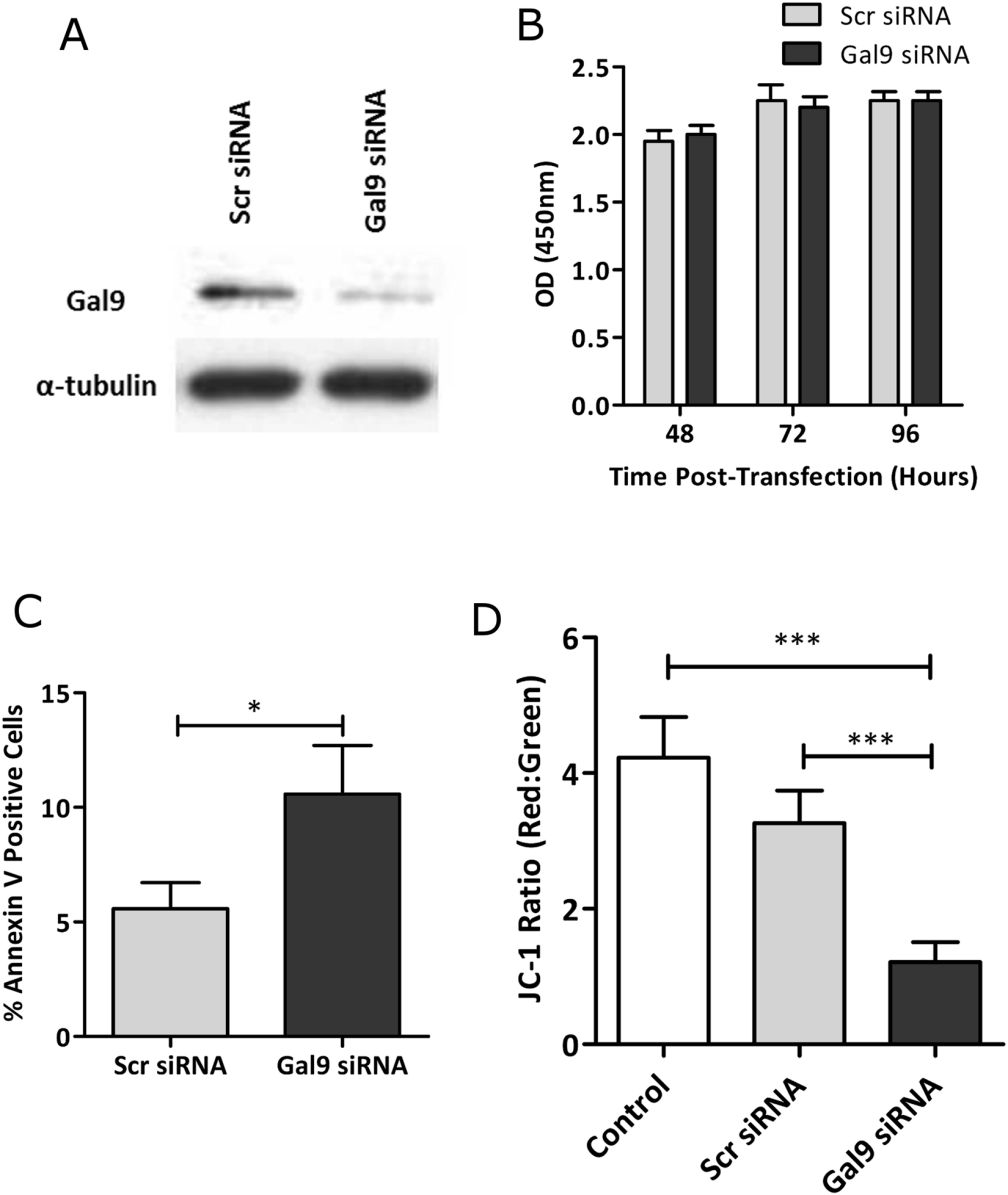
(**A**) Gal9 knock-down following siRNA transfection was confirmed by Western blotting. (**B**) Cell proliferation in the presence of TNF*α* and IL-1*β* (10 ng/ml of each) was shown to be unaffected by siRNA transfection. Data are mean ± SD. Apoptosis in each fibroblast population was measured by (**C**) annexin V staining and (**D**) JC-1 mitochondrial membrane depolarisation staining. *p < 0.05, ***p < 0.001. Full uncropped versions of the blots are shown in the supplementary data.

**Figure 5:**
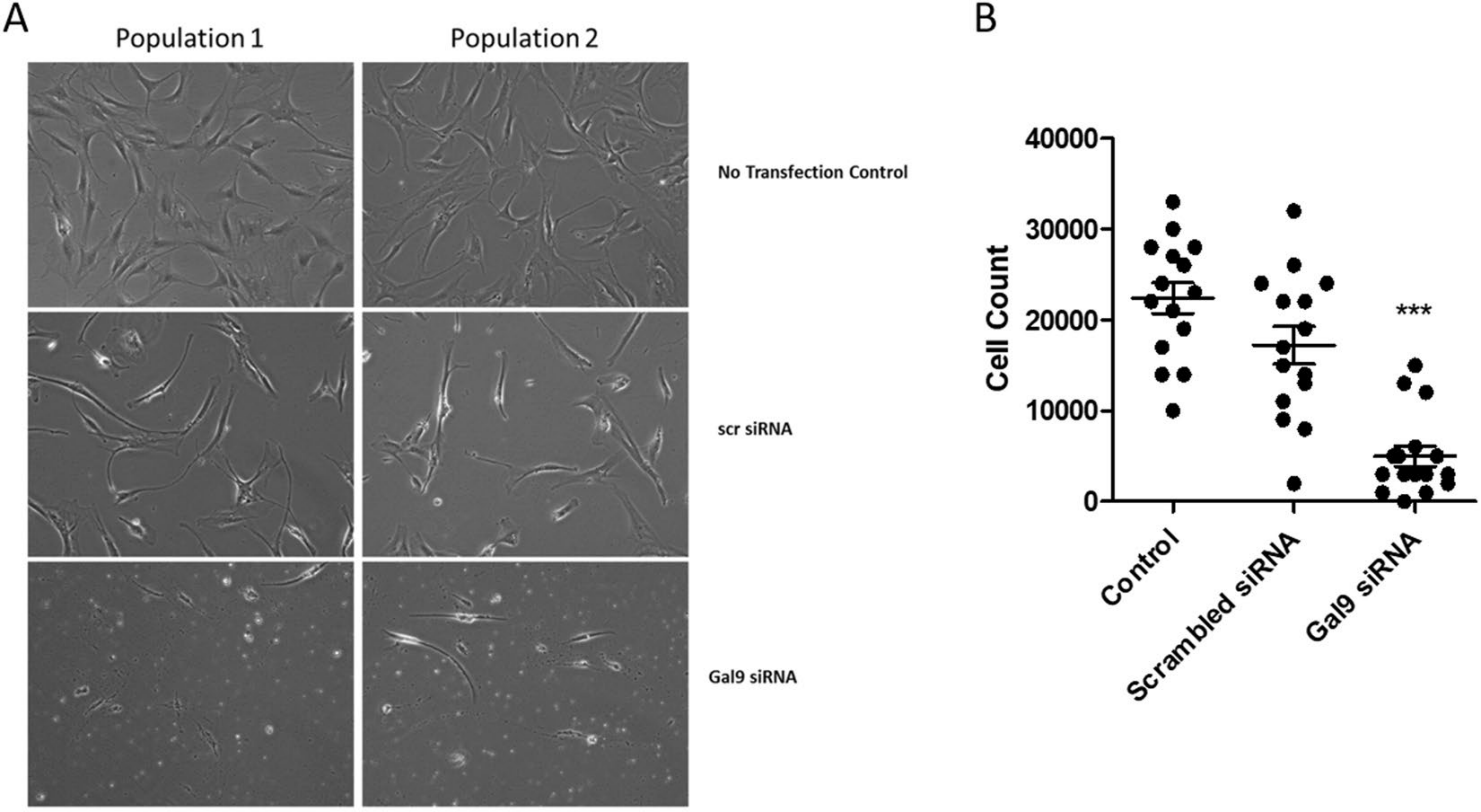
(**A**) Fibroblasts were visualised by light microscopy following transfection with scr siRNA, Gal9 siRNA or treatment with Lipofectamine only (no RNAi control). Original magnification x40. Images are representative of 2 populations from a total of 5 primary RA synovial fibroblast populations observed. (**B**) Cell counts of n = 5 primary human synovial fibroblast populations in a 12-well plate following Gal9 siRNA transfection. Data are pooled from three separate experiments. Statistical significance was determined using a one-way ANOVA with Tukey post-hoc test.***p < 0.001.

To determine whether the increased expression of Gal9 might affect synovial fibroblast proliferation and apoptosis RA synovial fibroblasts were transfected with Gal9 siRNA, or a scrambled siRNA (Scr) sequence and a control group of cells were left un-transfected. Effective silencing of Gal9 expression using RNA interference was confirmed by Western blotting (Fig. 4A). In order to assess whether knockdown of Gal9 affected fibroblast proliferation, XTT reduction to formazan was measured at 48, 72 and 96 h post Gal9 siRNA transfection. Gal9 knockdown was shown to have no effect on fibroblast proliferation stimulated with TNF*α* and IL-1*β* (10 ng/ml each) (Fig. 4B). AnnexinV binds to phosphatidylserine exposed on the cell membranes of apoptotic cells. Staining with annexin V-FITC showed greater binding of annexin V to the Gal9 siRNA transfected population compared to the scr siRNA population (Fig. 3C), suggesting that loss of Gal9 leads to increased apoptosis in these cells. This finding was confirmed using JC-1 staining to measure mitochondrial membrane depolarisation as a second measure of apoptosis, (Fig. 4D), consistent with increased apoptosis after Gal9 knock down. The Gal9 siRNA transfected fibroblasts also displayed visual signs of cell death 24 hours post-transfection with marked cell cytoplasm shrinkage, rounding up of cells and loss of classic fibroblast morphology (Fig. 5A).

## Discussion

Galectins 1 and 3 have previously been described as mediators of inflammatory processes^27–29^ where they act as chemoattractants for neutrophils, and facilitate recognition and killing of bacteria in pathogen-mediated inflammation^10^. In the present study we assessed galectin expression in fibroblasts from synovial tissue and matched non-inflamed skin of patients with RA and showed that expression of Gal9 was unique in being differentially increased in the synovial fibroblasts. Gal9 has been reported to be expressed at high levels within the RA synovium and previous reports have shown exogenous Gal9 to have a pro-apoptotic role within this tissue^25^. The very low level of Gal9 in skin fibroblasts in the present study suggests that the raised Gal9 could be a consequence of the local inflammatory environment. Fibroblasts play a central role in the “activated” synovium, recruitment of immune cells to the inflammatory milieu, and thus maintenance of persistent inflammation^1,30^. Their survival and proliferation within the inflamed joint may, therefore, represent a key early step in the transition to chronic inflammation. Knockdown of Gal9 by siRNA induced apoptosis in RA synovial fibroblasts, suggesting that endogenous Gal9, in contrast to exogenous Gal9^25^, protects against rather than induces apoptosis.

The induction of apoptosis following siRNA knockdown of Gal9 compared with the pro-survival effects of exogenous Gal9 shown previously by Seki and colleagues^25^ indicates that endogenous and exogenous forms of Gal9 play opposing roles in regulating cell death. In the RA synovium, fibroblast populations are maintained, in part due to the cytokine and chemokine repertoire produced by the inflammatory milieu. We have demonstrated here that activation of the TLR3 and TLR4 pathways in synovial fibroblasts lead to increased protection from apoptosis of synovial fibroblasts through induction of Gal9 expression. TLR expression in the RA synovium has been well characterised^31^ and it is known that TLRs in concert with pattern recognition receptors (PPRs), in particular nucleotide-binding oligomerisation domain 2 (NOD-2) activate synovial fibroblasts and promote and maintain inflammatory mediator expression^32^.

In the current study, IFN*γ* was the most potent inducer of Gal9 expression (Fig. 3B). Additionally, our data suggest that an inflammatory environment containing IFN*γ* would profoundly upregulate the short isoform of Gal9. Therefore, it could be that this short isoform is of functional significance in protection of synovial fibroblasts against apoptosis during inflammation and may represent an important therapeutic target.

We have shown that endogenous Gal9 expression also maintains the fibroblast population through protection against apoptosis, and thus contributes directly to the maintenance of persistent inflammation within the RA joint. Understanding the endogenous and exogenous roles of Gal9 is integral to understanding how to manipulate it for use as a therapeutic target. Endogenous Gal9 has been implicated in prevention of malignant tumour progression through disruption of CD44-hyaluronan interactions^33^, acting as a potent IgE antagonist preventing degranulation of mast cells^34^ and as an inducer of immunosuppressive macrophages which ameliorate T cell-driven inflammation^35^. Exogenous Gal9, however, has been shown to induce apoptosis in synovial fibroblasts, T cells^25,36^ and other immune cell lineages^18^ to prevent Th17 differentiation^2^ and to reduce inflammation in psoriasis patients^37^. Unlike Gal3, Gal9 is largely intracellular *in vivo* due to its lack of a signal peptide for secretion^38^. There is currently no evidence that fibroblasts are able to secrete the Gal9 which they produce, unlike T cells which readily secrete it upon T cell receptor (TCR) stimulation^39^. Therefore, it is possible that intracellular Gal9 effects may dominate extracellular Gal9 effects that are generated through cell surface receptors such as TIM3. The nature of Gal9 intracellular function could be similar to that observed for intracellular Gal3, which is known to interact with members of the anti-apoptotic Bcl-2 family^40^. There are, therefore, two possible means of exploiting Gal9 as a therapeutic: firstly removing blockade of apoptosis through blockade of endogenous, intracellular Gal9. Secondly, through administration of exogenous Gal9 which can signal through membrane bound receptors to induce apoptosis. We conclude that Gal9 represents an important and novel target for treatment of RA and potentially other auto-inflammatory diseases.

## Methods

### Galectin expression in synovial tissue and fibroblasts

RA synovial tissue was obtained from biopsies from RA patients recruited from Sandwell and West Birmingham Hospitals NHS Trust and University Hospitals Birmingham NHS Foundation Trust UK. All patients fulfilled the 1987 ACR classification criteria for RA with symptom duration of greater than 3 months, were CCP+, RHF+ and underwent synovial biopsy of the knee. All patients provided written, informed consent (West Midlands Black Country Research Ethics Committee Approval 07/H1203/57). Frozen RA tissues were cut in 6 *μ*m thick sections, fixed with acetone and frozen prior to use. Slides were washed in Phosphate Buffered Saline (PBS), incubated for 30 min in 10% Horse serum/PBS and incubated overnight at 4^*circ*^C with primary antibodies to Galectin-9 (Abcam, Cambridge, UK), Fibroblast Activation Protein, FAP (Invitrogen, Waltham, MA, USA, clone F11-24) and Podoplanin (Invitrogen, clone NZ-1.3). After washing with PBS, slides were incubated with secondary antibodies anti-mouse IgG1 Alexa 546 (Life Technologies, Paisley, Scotland), anti-rabbit IgG Alexa 488 (Life Technologies) and anti-rat IgG Alexa 647 (Jackson ImmunoResearch, Cambridge, UK) for 30 min, counterstained with DAPI (Life Technologies) and mounted in aqueous mounting media. Tissues were imaged using a Zeiss LSM 780 confocal microscope and processed using ZEN black (ZEISS, Cambridge, UK). Fibroblasts were isolated by explant culture from synovial tissue and skin from the same patients as previously described^41^ and grown to confluence to determine expression of galectins 1, 3, 4, 8 and 9 by real-time PCR. All patients provided written, informed consent (West Midlands Black Country Research Ethics Committee Approval 07/H1204/191).

### Real-Time PCR

RNA was prepared using an RNeasy Mini Kit (Qiagen, Manchester, UK) and reverse transcription was carried out using Superscript Vilo (Life Technologies, Paisley, UK) according to the manufacturer’s instructions. Real-time PCR was carried out using FAM-labelled TaqMan® Human Gene Expression assays for the galectins of interest as well as IL-1*β*, IL-4, IL-6, IL-17, IFN*γ*, TNF*α*, TGF*β* and *β*-actin control (Life Technologies, Paisley, UK). All samples were analysed using a 7900HT real-time PCR machine (Life Technologies, Paisley, UK). Data were expressed as ΔΔCt values.

### Cell stimulation

RA synovial fibroblasts were seeded into 24 well plates and incubated at 37 °C, 5% CO_2_ until confluent. The culture medium was replaced with 500 *μ*l fresh complete RPMI 1640 medium (10% FCS, 400 *μ*M L-glutamine, 2,000 U/ml penicillin, 2 mg/ml streptomycin, 1% v/v NEAA, 1% v/v sodium pyruvate, all from Sigma Aldrich, Gillingham, UK) prior to treatment with 10 ng/ml of each of the cytokines IL-1*β*, IL-17, IFN*γ*, TNF*α*, TGF*β*, IL-4 (all R&D Systems, Abingdon, UK), or IL-6 (Peprotech, New Jersey, USA) (20 ng/ml), or TLR agonists 300 ng/ml bacterial lipoprotein (bLP; InvivoGen, San Diego, US), 10 *μ*g/ml poly(I:C) (InvivoGen, San Diego, US), or 100 ng/ml LPS (List Biological Laboratories, Campbell, USA). The cells were incubated for 24 hours at 37 °C, 5% CO_2_ after which RNA was extracted for Gal9 expression as determined by Real time PCR.

### Western blotting

Synovial fibroblasts were washed with PBS and lysed with SDS loading buffer (0.125 M Tris pH 6.8, 20% glycerol, 2% SDS, 5% 2-mercaptoethanol and 25 mg/ml bromophenol blue). Lysates were then heated for 10 minutes at 100 °C. Samples were run on a 12% SDS-PAGE gel followed by transfer to PVDF membrane. Gal9 was detected using an anti-Gal9 primary antibody (GalPharma) and anti-mouse secondary antibody (ThermoFisher, Uk). Blots were developed with ECL before transfer to x-ray film (Kodak). Densitometry was determined using ImageJ (NIH).

### Galectin 9 silencing

Synovial fibroblasts at passage 3–6 were seeded into 6-well culture plates and incubated in complete RPMI 1640 at 37 °C to 50% confluence. Fibroblasts were transfected using Lipofectamine 2000 (Life Technologies, Paisley, UK) following the manufacturers protocol. siRNA was used at a final concentration of 90 nM for both Scr and Gal9.

### Fibroblast Proliferation Assay

Fibroblast proliferation was assessed using XTT (Cell Proliferation Kit; Biological Industries, Kibbutz Beit-Haemek, Israel) following Gal9 knockdown. XTT is reduced to formazan by live cells and the intensity of absorption at 450 nm is directly proportional to the number of live metabolically active cells present^42^. Fibroblasts were cultured in 96-well plates at a density of 8 × 10^4^ cells/100 *μ*l culture medium. As a positive control, fibroblasts were stimulated with 10 ng/ml TNF*α* and 10 ng/ml IL-1*β*. Cell proliferation was assessed at 48, 72 and 96 h post-stimulation. XTT was prepared according to the manufacturer’s instructions and was added to the fibroblasts. Fibroblasts were incubated for 3 h in the presence of XTT at 37 °C and the plate was read at 450 nm with a reference wavelength of 620 nm.

### Fibroblast Apoptosis Assays

Synovial fibroblast apoptosis was assessed using two methods, annexin V binding^43^ and JC-1 staining^44^. For Annexin V staining fibroblasts were trypsinised and resuspended in RPMI 1640 and combined with the cell culture supernatants containing non-adherent dead cells and washed in PBS (300 *xg*, 5 minutes). The resulting pellet was resuspended in annexin V buffer (10 mM HEPES, pH7.4; 140 mM NaCl; 2.5 mM CaCl2) and stained with anti-annexin V-FITC and propidium iodide (PI) (eBioscience). Annexin V positive cells were detected using a CyAn ADP flow cytometer (Beckman Coulter, High Wycombe, UK).

For JC-1 staining fibroblasts were stained with 5,5′,6,6′-tetrachloro-1,1′,3,3′-tetraethyl-imidacarbocyanine iodide (JC-1) (Sigma Aldrich, Dorset, UK). In live cells, JC-1 exists in both its monomeric and aggregated forms which emit fluorescence at 530 nm (green) and 590 nm (red). In apoptosing or dead cells, only the monomeric form exists. RA synovial fibroblasts transfected with Gal9 siRNA, Scr siRNA and the control group were stained with JC-1 following the manufacturer’s instructions, and the shift from red to green fluorescence was measured using an Accuri C6 flow cytometer (Becton Dickinson, Oxford, UK).

### Statistical Analysis

Non-parametric distribution was assumed for all assays. For comparison of two groups the Mann-Whitney U test was used. Analysis of Gal9 expression in multiple groups of fibroblasts was performed with Kruskal-Wallis one-way analysis of variance and Dunn’s post-test. All analyses were carried out using the Graphpad Prism 5 statistical software package.

## Electronic supplementary material

Supplementary Figures
