# Supplementary material for: Endogenous Galectin-9 Suppresses Apoptosis in Human Rheumatoid Arthritis Synovial Fibroblasts
Source: Sci Rep. 2018 Aug 27;8:12887. doi: 10.1038/s41598-018-31173-3 (PMC6110759; doi:10.1038/s41598-018-31173-3)
Supplement: Supplementary file 1 — Supplementary Figures [file 41598_2018_31173_MOESM1_ESM.docx]

**Endogenous Galectin-9 Suppresses Apoptosis in Human Rheumatoid Arthritis Synovial Fibroblasts**

**Mark J Pearson**^1,4+^**, Magdalena A Bik**^2+^**, Caroline Ospelt**^3^**, Amy J Naylor**^2,4^**, Corinna Wehmeyer**^2,4^**, Simon W Jones**^2,4^**, Christopher D Buckley**^2,4^**, Steffen Gay**^3^**, Andrew Filer**^2,4^**, and Janet M Lord**^2,4*^

^1^Aston Medical School, Aston University, Birmingham B4 7ET

^2^NIHR Birmingham Biomedical Research Centre, University of Birmingham, Birmingham, B15 2TT UK

^3^Center of Experimental Rheumatology, Department of Rheumatology, University Hospital of Zurich, CH-8091 Zurich, Switzerland

^4^MRC-Arthritis Research UK Centre for Musculoskeletal Ageing Research, Institute of Inflammation and Ageing, University of Birmingham, Birmingham, B15 2TT, UK.

^*^Corresponding Author: Professor Janet M Lord (J.M.Lord@bham.ac.uk)

^+^These authors contributed equally to this work


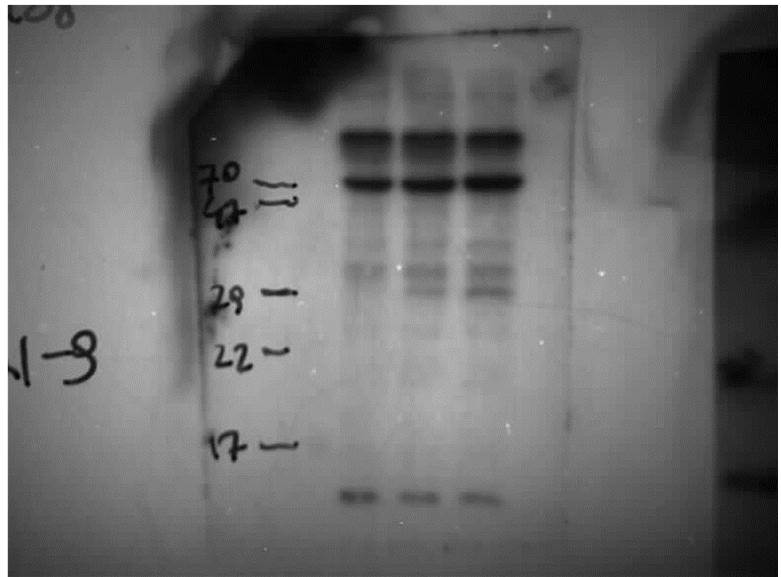


Supplementary Figure 1: Full size image of the Western blot shown in Figure 3c, top panel. The two isoforms of Gal9 can be observed at 35 and 39KDa respectively. Lane 1: Molecular weight marker; Lane 2: no stimulation of synovial fibroblasts; Lane 3: synovial fibroblasts +IFNγ for 24h; Lane 4: synovial fibroblasts +IFNγ for 48h. IFNγ added at 10ng/ml.


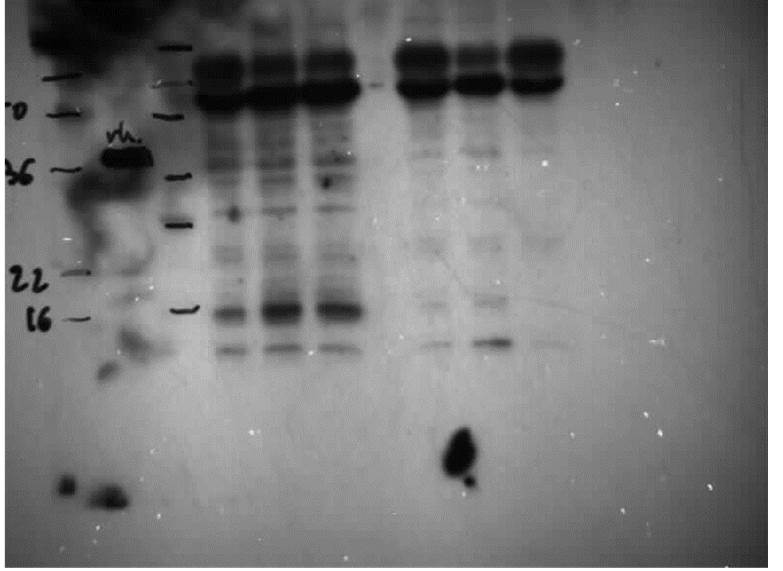


Supplementary Figure 2: Full size image of the Western blot shown in Figure 3c, bottom panel. The two isoforms of Gal9 can be observed at 35 and 39KDa respectively. Lane 1: Molecular weight marker; Lane 6: no stimulation of synovial fibroblasts +anti-IFNγ; Lane 7: synovial fibroblasts +IFNγ, +anti-IFNγ for 24h; Lane 8: synovial fibroblasts +IFNγ, + anti-IFNγ for 48h. IFNγ added at 10ng/ml.
